# Development of *CryoVR*, a virtual reality training system for hands-on cryoEM operations

**DOI:** 10.1107/S2059798322005654

**Published:** 2022-06-27

**Authors:** Jiahui Dong, Daoyi Li, Kadir Ozcan, Dayu Wan, Wen Jiang, Yingjie Chen

**Affiliations:** a Computer Graphics Technology, Purdue University, Knoy Hall Room 363, 401 North Grant Street, West Lafayette, IN 47907, USA; b Department of Biological Sciences, Purdue University, 915 W. State Street, West Lafayette, IN 47907, USA

**Keywords:** *CryoVR*, cryoEM, virtual reality, virtual reality training, human–computer interaction, structural biology

## Abstract

Cryogenic electron microscopy (cryoEM) has emerged as a revolutionary method for solving high-resolution structures and studying the dynamics of macromolecular complexes and viruses in near-native states. However, the availability of the equipment, and the time and cost needed for training, severely limit the opportunities for training. To solve these problems, a virtual reality-based training system, *CryoVR*, has been developed to prepare trainees before operating real-world cryoEM equipment. This paper describes the design and assessment of *CryoVR* (available at https://www.purdue.edu/cryoVR), which helps users learn cryoEM experimental procedures in a virtual environment, allowing immersive training with step-by-step tutorials with vivid visual, audio and text guidance. Implemented as a training step before a novice user interacts with the expensive real-world cryoEM equipment, *CryoVR* can help users to become familiar with hands-on operational procedures through multiple training modules and earning certificates after passing the built-in Exam mode. Qualitative evaluation and feedback of *CryoVR* from users with various levels of cryoEM experience indicate the substantial value of *CryoVR* as a tool for a comprehensive cryoEM procedural training.

## Abstract 2

This paper describes the design and assessment of *CryoVR*, a virtual reality-based training system for cryoEM devices. *CryoVR* provides a unique learning opportunity by giving users experience of cryoEM experimental procedures through a safe and accessible virtual environment.

## 1. Introduction

Cryogenic electron microscopy (cryoEM) has emerged as a revolutionary method for solving high-resolution structures and studying the dynamics of biological macromolecular complexes and viruses in near-native states (Jiang & Tang, 2017 ▸; Callaway, 2015 ▸). The rapidly increasing number of near-atomic resolution structures solved using single-particle cryoEM has generated the data needed to understand a variety of fundamental biological processes (Sirohi *et al.*, 2016 ▸; Wrapp *et al.*, 2020 ▸; Yan *et al.*, 2020 ▸). The cryoEM resolution revolution has attracted attention from researchers in various biomedical fields, and thus there is a crucial need to expand the training capabilities of cryoEM facilities around the world. However, the training capacities of these facilities are limited due to the need for repetitive practices on the instruments by the trainees, limited cryoEM instruments and staff availability, and cost (Alewijnse *et al.*, 2017 ▸). The complex practical procedures required by cryoEM pose a steep learning curve to new users, and the challenges in offering fast, high-capacity training are considerable hurdles in cryoEM training. Moreover, a clear demonstration of safety procedures is crucial to reduce the potential risk of operating the equipment. Effective cryoEM training requires a combination of theory, observation and hands-on operation under close supervision from highly trained experts. These limitations in training have been well recognized and there are many efforts to address these needs. For example, the NIH Common Fund Transformative High Resolution Cryo-Electron Microscopy program (https://commonfund.nih.gov/cryoem) has funded multiple national cryoEM centers and cryogenic electron tomography (cryoET) centers to provide both data-collection and user-training services. The NIH cryoEM program has also funded multiple curriculum-development teams to create new training materials spanning a wide range of approaches from web-based lectures on cryoEM (available at https://cryoem101.org/), in-depth theoretical understanding of principles (available at https://cryoemprinciples.yale.edu/), a comprehensive collection of videos on theory and practical operations (available at http://cryo-em-course.caltech.edu/) and, to our team, a virtual reality (VR) augmented training system named *CryoVR* for hands-on operations (Gonzalez *et al.*, 2019 ▸).

Since the first consumer-level head-mount display (HMD) for VR was brought to the market in 2013 by Oculus (https://www.oculus.com/compare/), VR has become accessible to the public for gaming, education and research (Jensen & Konradsen, 2018 ▸). Modern VR technology has made the virtual experience much more realistic and affordable. The high-fidelity simulated sensations of visual, audio and touch by VR devices can create the feeling of presence in a virtual environment for the user. VR training can reduce training costs, especially when traditional training requires experienced expert trainers and can suffer from the breakdown of devices used during training (Carlson *et al.*, 2015 ▸). VR has been widely adopted in simulation training for critical operations such as flight (Yavrucuk *et al.*, 2011 ▸) and medical surgery (Lam *et al.*, 2013 ▸) as it enables knowledge and skills to be acquired in an efficient, consistent and economical manner. In a systematic review of 34 studies from 2016 to 2018 on applications of VR in higher education, it was found that the true potential of VR is to provide the students with the ‘learn by doing’ experience, which is often difficult to implement in traditional lectures.

In this paper, we introduce the development of *CryoVR* (*CryoEM Virtual Reality*), a VR training system that is dedicated to training users in the sample grid-preparation procedures of single-particle cryoEM. It contains four groups of modules: Grid Glow Discharging (easiGlow), Plunge Freezing (ThermoFisher Vitrobot and Gatan CP3), AutoGrid Clipping and AutoGrid Loading.

## 2. VR augmented training theory

Recent studies have found that VR training is especially suitable for delivering procedural knowledge and declarative knowledge. Procedural knowledge refers to the knowledge of how to perform a specific task, for example procedures of operating an instrument. While current VR technology still cannot vividly simulate fine haptic feedback and is not able to train fine motor skills of the hands and body during operation, researchers have found that in terms of procedural knowledge training a VR environment can be as effective as a real environment (Smith *et al.*, 2016 ▸). For example, military tank-maintenance work can be learned in a virtual environment as well as in a real-world training environment based on the success rate, time and the amount of guidance needed from an instructor (Ganier *et al.*, 2014 ▸). It was found that in a VR environment using a ghost virtual trainer in a first-person viewpoint (called Just Follow Me; Yang & Kim, 2002 ▸) can produce a training and transfer effect at least as good as in the real world. This Just Follow Me approach can transfer proprioceptive information more directly and with high fidelity from a trainer to a trainee. The effect of VR on learning can be explained by multiple cognitive theories (Dale, 1969 ▸). The experience theory demonstrates that learners can only remember 10% of what they read, but up to 90% if they can perform actions during a training period, suggesting that practical experience is vital for learning procedural knowledge (Anderson, 1982 ▸). These findings suggest that VR is promising for procedural learning by providing learners with the ‘learn by doing’ experience. Based on embodied cognition theory, embodied learning has become an important part of modern learning practices (Foglia & Wilson, 2013 ▸; Price *et al.*, 2009 ▸). An embodied learning scheme can be a great expansion of conventional training (Abrahamson, 2014 ▸) because it can provide the engagement of the physical body in multi-modal learning experiences which may involve bodies, minds and social beings (Nguyen & Larson, 2015 ▸; Wilson, 2002 ▸). Andrade and coworkers suggested that attention to gestures and movements in the design of learning environments can improve learning (Andrade *et al.*, 2017 ▸). Designing instructions to account for the body can legitimize and mix new modalities and sets of intellectual resources for learning (Enyedy *et al.*, 2015 ▸). As VR training typically involves the embodied learning activities by providing the trainees with virtual hands-on experiences, VR has the potential to leverage embodied cognition and can boost learning outcomes (Kosmas *et al.*, 2018 ▸; Macedonia *et al.*, 2011 ▸; Repetto *et al.*, 2015 ▸).

## 3. *CryoVR*: motivations and goals

CryoEM projects consist of multiple steps with very different characteristics; for example, sample grid preparation and data collection require hands-on operation of various instruments, while image processing and 3D reconstruction require the running of complex computer programs. Training for these distinct tasks is best served by multiple approaches or media (Lyumkis, 2019 ▸). Much of the training in theoretical knowledge and computational tasks can be implemented through in-person or remote classes, workshops or self-learning using a combination of PowerPoint slides, video recordings, live presentations and reading of manuals and protocols. However, training tasks that teach the users how to properly operate various instruments, for example plunger freezers and electron microscopes, require physical access to the instruments. A common feature of these tasks is that there are multiple steps to be performed in a particular sequence, and the learning requires hands-on operations of the instruments under the close supervision of an expert. These hands-on operations require exceptional cognitive skills and precise execution of correct procedures backed up by sufficient domain knowledge. These training tasks can be time-consuming, for example, observation of a 2–4 h demo session for cryo-grid loading and low-dose EM imaging followed by multiple supervised 2–4 h practice sessions before certification by the facility for independent work (Eng *et al.*, 2020 ▸). This hands-on training is essential, but also challenging, as it is restricted by the availability of both the trainer and the instrument, often leading to long waiting times and potentially many weeks between sessions. To master the range and complexity of skills needed for cryoEM, trainees need to practice repeatedly to accumulate sufficient ‘mileage’ to achieve skill mastery. Any significant time away from the instruments requires additional time to refresh the skills. Inexperienced trainees, when practicing without the close supervision of experts, may make ‘rookie’ mistakes that can lead to instrument damage, further constraining the training and data-collection capacity of cryoEM facilities. For example, dropping an unsecured grid or clip ring into the stage or column from a side-entry holder can result in a downtime of 1–2 weeks, which not only impacts instrument availability but also incurs expensive service costs beyond warranty. The availability and cost of occupying cryoEM instruments and the trainer time and risk of damaging the instruments often render these training tasks a bottleneck in the overall training of new users. The ongoing COVID-19 pandemic has also unexpectedly further reduced access to cryoEM facilities and increased the health risks of one-on-one training.

Building on the knowledge of VR in effective training of procedure knowledge and its potential for training operations of devices with limited access, such as the sample-preparation devices and electron microscopes involved in cryoEM, we have thus developed a VR-based interactive training system, *CryoVR*. The significance of *CryoVR* lies in its ability to provide a virtual, engaging, self-paced hands-on training environment for users to familiarize themselves with the instrument and operation protocols before accessing the physical instruments. It not only can serve as an effective pre-training system for new users, but also can help an infrequent user to warm up their skills before returning to real operations on cryoEM instruments. Virtual mastery of operational procedures will not only significantly reduce the per-trainee time necessary for physical instrument and trainer supervision, but also should be very effective in reducing ‘rookie’ errors that might damage the physical instruments.

**Figure 1:**
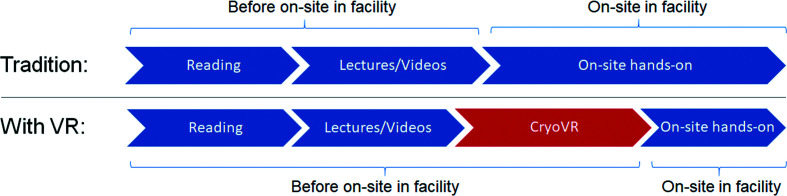
The position of *CryoVR* in the cryoEM training pipeline.

Due to the complexity and interdisciplinary nature of cryoEM, the learning curve for cryoEM is steep and training is time- and resource-demanding. A typical traditional training pipeline can be roughly divided into three stages (Fig. 1 ▸): a trainee can start self-learning by going through the literature or taking a course to learn the theory and protocols for each of the specific tasks. There are already many excellent resources for users to go through these training steps. These teaching materials are exhaustive but have an inadequate level of interaction from the learner side. In our training system, *CryoVR*, the users can benefit from the immersive VR environment and the gamification features to improve their learning efficiency.

It should be pointed out that cryoEM tasks consist of two major types of tasks: hands-on operations and computer screen-focused operations (Nogales & Scheres, 2015 ▸). For example, the loading/unloading of sample grids to/from the autoloader of a Titan Krios CryoTEM primarily uses hands-on operations, while the instrument alignment and automated data collection using software such as *Leginon*, *SerialEM* or *EPU* *etc.* are primarily performed through the mouse, keyboard and computer screens. Only the hands-on operations are well suited for *CryoVR*, while computer screen-focused interactions are more suited by other training approaches that are beyond the scope of *CryoVR*. As an exception, a small amount of computer-screen interactions, for example for the Vitrobot, can be simulated well by *CryoVR*. We would like to emphasize that *CryoVR* will augment but not replace traditional training approaches. In the VR training pipeline (Fig. 1 ▸), *CryoVR* is intended as a pre-training step to familiarize trainees with the instruments and operational procedures using the virtual instruments in *CryoVR* before starting actual hands-on training on the physical instruments in the cryoEM facility. By pre-training on the virtual instruments and achieving virtual mastery of the operation procedure, trainees are expected to need significantly fewer orientation sessions on the instrument and can instead focus on polishing the fine touches of manipulating the physical objects that current VR technology is not able to faithfully reproduce. The final stage of practice on physical instruments will still be essential, but *CryoVR* pre-training will help to significantly reduce the amount of time needed. This pre-training provides multiple benefits to the trainees, including faster completion of training and less expense to cover the instrument usage and trainer time. For trainers and cryoEM facilities, the benefits include an increased training capacity to grow the skilled workforce, more instrument time for data collection and potentially less damage and downtime of the instruments caused by ‘rookie’ errors. Of course, the final certification by the trainer for independent operation of the physical instruments will continue to be performed on the physical instruments. Considering the strengths and limitations of VR training, we set the following learning objectives for *CryoVR*.

(i) Demonstrate familiarity with the sequence of operations needed for major cryoEM instruments prior to one-on-one hands-on training on physical instruments.
(ii) Demonstrate a reduced training time on physical instruments needed to obtain approval from the trainer for the independent operation of cryoEM instruments.
(iii) Demonstrate a conceptual understanding of safety procedures to avoid key errors and know how to respond appropriately in the event of errors.

## 4. *CryoVR* modules and features

### 4.1. Training modules

Currently, *CryoVR* covers the early-stage tasks of a single-particle cryoEM project: from grid glow discharging, plunge-freezing and clipping the autogrids to loading the grids into the TEM column. Each of the tasks is implemented in a separate module that can be individually installed and played. The executables of the *CryoVR* modules are freely available at https://www.purdue.edu/CryoVR and the source code and models are freely available at https://github.com/CryoVR.

#### 4.1.1. Grid Glow Discharging

**Figure 2:**
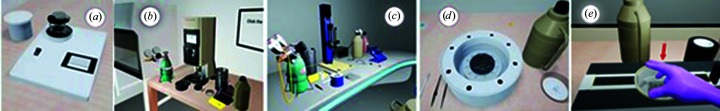
*CryoVR* modules. (*a*) Grid Glow Discharging (easiGlow); (*b*, *c*) Plunge Freezing with TFS Vitrobot Mark IV (*b*) and Gatan CP3 (*c*); (*d*) TFS AutoGrid Clipping; (*e*) TFS AutoGrid Loading.

This module simulates glow discharging of cryoEM grids, which is frequently used to remove surface contamination and render the grid surface hydrophilic before samples are applied and plunge-frozen (Fig. 2 ▸ *a*).

#### 4.1.2. Plunge Freezing

We have created two modules to simulate two plunger-freezing devices for sample grid preparation: the ThermoFisher Scientific (TFS) Vitrobot Mark IV (Fig. 2 ▸ *b*) and the Gatan CP3 (Fig. 2 ▸ *c*).

#### 4.1.3. Autogrid Clipping

This module simulates how TFS autogrids are assembled in a dedicated workstation by seating cryoEM grids over a clip ring and then locking the grid and clip ring together with a C-shaped spring or C-clip (Fig. 2 ▸ *d*).

#### 4.1.4. Autogrid Loading

This module teaches the users how to insert the frozen autogrids into the autoloader cassette and then load the nanocab with the cassette into the autoloader of the cryoTEM (Fig. 2 ▸ *e*).

The virtual environment of these *CryoVR* modules simulates a real laboratory environment. In *CryoVR*, the user can walk around and look in arbitrary directions. At the start of each module, the user is positioned at the center of the virtual laboratory and faces the target cryoEM device of the module. In general, each module contains a workbench, the device related to the training and all necessary tools during the training procedure.

### 4.2. Training modes

#### 4.2.1. Tutorial mode

**Figure 3:**
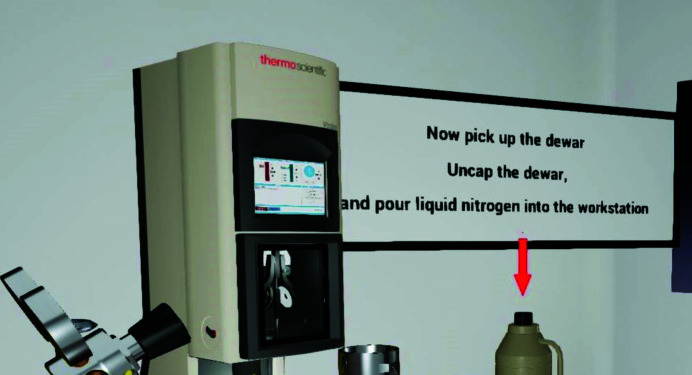
Tutorial mode of the Vitrobot training module in *CryoVR*. Voice, text/visual user interface and video instructions guide the user to learn the experimental procedures.

For those who are not familiar with experimental procedures, a Tutorial mode with fully guided text, sound and visual instructions is both useful and necessary. The instructions are designed to clearly demonstrate the detailed operation steps (Fig. 3 ▸), as well as showing potential mistakes that users may make during their experiments. As a result, users will gain comprehensive experience during VR training that will carry forward to training on the physical instruments. Additionally, there are video clips of expert operations embedded in *CryoVR* for cross-referencing. These modules can record a the actions of a user and play back the recordings to help users improve their learning retention and become aware of mistakes that they may have made.

#### 4.2.2. Exam mode

We aim at conferring the users of *CryoVR* modules with sufficient skills necessary for the use of the instruments and their operational procedures to reduce the time needed for their final on-site training on physical instruments. To assess learning outcomes, a final examination will be critical to evaluate user skills. In our survey, a majority of respondents (74.3%) considered the *CryoVR* Exam mode important. Currently, we score the user’s operations against a standard operation procedure, with each operation step specified by the instrument parts or ancillary devices to be operated, the starting/ending positions, allowable time *etc*. In practice, some alternative operations or sequences of operations in a procedure can also be acceptable for a real experiment. We have consulted with cryoEM operation experts to incorporate acceptable variations in *CryoVR* scoring criteria. To ensure mastery and avoid future mistakes on physical instruments, we require a perfect score (100 points) in the Exam mode to complete a *CryoVR* training module. We have found that most novice users could pass the Exam mode after just a few passes through the modules.

### 4.3. Standard operation procedures (SOPs)

To better coordinate the training efforts among the NIH-funded national CryoEM centers and curriculum developers (with *CryoVR* being one of the curriculum developers), these teams are currently collaborating to establish SOPs in the different cryoEM tasks which *CryoVR* has implemented in the corresponding modules. At the same time, a merit badge system is designed to certify the training outcome that will be honored across the centers. In *CryoVR*, once the user passes the Exam mode of the certain module, a certificate will be issued to the user, recorded in our online database and can be verified by trainers.

### 4.4. Safety training

In any laboratory training, safety is a top priority. We note the importance of including the same safety training within our *CryoVR* modules. There are multiple potential safety risks related to the use of cryoEM instruments and operations, for example, fire hazard from ethane gas, cold burns from liquid nitrogen *etc*. We consider VR an ideal environment for new users to familiarize themselves with potential dangers in using instruments and reagents without risking harm to themselves or others or damaging expensive equipment and samples. In *CryoVR*, we include both general laboratory safety and cryoEM-specific safety to ensure that the users understand risks, know how to avoid risks and know how to properly respond to these risks to protect themselves and the equipment. We have developed the relevant models and instructed users to begin all laboratory procedures by putting on gloves, a laboratory coat and safety goggles. By reinforcing this behavior in VR, we expect users to always think about safety and protection during real-world operations. As the VR game engine can return all necessary data, including time, position, rotation and status, it is possible not only to detect whether users follow the safety procedures but also to simulate the disastrous consequences of missing the safety procedures, for example, a fire will be caused if the user has forgotten to close the valve of the ethane gas tank during a plunge-freezing VR session. The dramatic virtual experience of disasters in *CryoVR* should help ensure the users to consciously follow the safety procedures and avoid safety accidents in real-world operations.

### 4.5. Supported VR platforms

**Figure 4:**
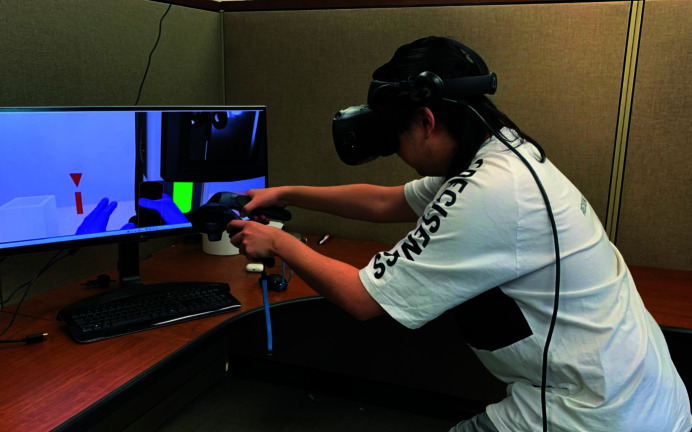
A user performing virtual plunge-freezing in *CryoVR* with an HTC Vive device.

*CryoVR* supports major VR devices such as the HTC Vive series (Borges *et al.*, 2018 ▸) and Oculus Quest series (https://www.oculus.com/compare/). The user will use two controllers of these VR devices as their virtual hands to interact with the virtual objects (Fig. 4 ▸). We recommend using the high-end devices from these series, for example HTC Vive Pro or equivalent, due to their superior performance at relatively affordable cost ($499–1199). Accurate tracking, high video resolution (2800 × 1600) and refresh rate (≥72 Hz) and a wide field of view (110°) are all crucial to ensure the realism and accuracy of VR training (Niehorster *et al.*, 2017 ▸). With its flexibility and economy, the Oculus Quest series is another good option. We have developed specific *CryoVR* versions that perform better with an Oculus platform. These high-end consumer-level products have been widely used in serious business applications, such as hazardous situation training and medical surgery training.

## 5. Design considerations of *CryoVR*

Presence and realism are key factors that determine whether a user can have a satisfactory experience in a virtual environment and are vital to the success of VR training (Hays & Singer, 1989 ▸; Bowman & McMahan, 2007 ▸). Realistic surroundings and interactions with virtual objects are especially important elements when designing VR applications for higher education (Radianti *et al.*, 2020 ▸). ‘Immersion’ and ‘presence’ objectively measure a simulation environment. There are many key factors related to hardware, for example, a wide field of view, short reaction latency and high display resolution and refresh rate (Bowman & McMahan, 2007 ▸; Hoffman *et al.*, 2006 ▸; Meehan *et al.*, 2003 ▸; McMahan *et al.*, 2012 ▸). Researchers and hardware manufacturers have devoted huge efforts to improving VR devices and have made consumer-level VR headsets and controllers suitable for simulating daily physical activities (Borges *et al.*, 2018 ▸; Desai *et al.*, 2014 ▸). While *CryoVR* cannot improve hardware, we have made a great effort on the design and software-development side to make simulations high fidelity. More specifically, we focused on perceptual (for example visual and auditory) fidelity and interaction fidelity, as these significantly affect performance and subjective judgments of engagement and usability (McMahan *et al.*, 2012 ▸; Rogers *et al.*, 2019 ▸).

### 5.1. Accurate hand gestures for interactions with devices

**Figure 5:**
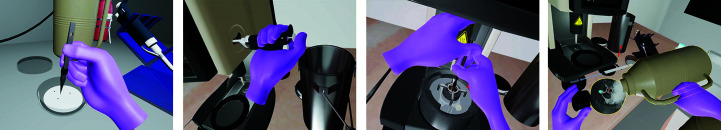
Different hand gestures tailed to operations for different virtual objects in *CryoVR*.

In cryoEM, there are some devices with complicated structures that require specific hand gestures to handle them in real life. To make the virtual operations realistic and to educate trainees in the proper way of handling an object or instrument, we carefully examined the standard modes of manipulating various objects and implemented corresponding hand gestures for each instrument (Fig. 5 ▸). These hand gestures largely cover various forms of grabbing, twisting, pressing and holding various objects. When a user’s virtual hand is close to the object, the hand gestures in VR will automatically change to the matching gesture at an appropriate pose to interact with the object. These gestures help to provide more intuitive interactions in *CryoVR*.

We conducted a human subject study to evaluate how *CryoVR* users react to virtual hand gestures in *CryoVR* using subjects outside the bioscience domain. There could potentially be some extent of cognitive conflicts between the user’s real hand gestures (always holding controllers with both hands) and their perceived hand gestures in VR (for example twisting a handwheel to open an ethane gas tank or picking up a grid using tweezers). In our study, the users did not report such conflict or inconsistency between their real hand gestures and perceived hand gestures. They feel the virtual hand natural as they are working with specific gestures. We then asked the users to pick up real devices (pipettes and tweezers) and to use them. Although none of these nonbiology users had ever used or even seen these devices in the real world, they could subconsciously properly use these devices. Apparently, the sense of perception can override feelings from their hands, and what was seen in VR influenced the trainee in the real world.

### 5.2. Visual and auditory fidelity

Visual realism is crucial for a user to feel realism and immersion in a VR environment since visual realism enhances realistic responses (Slater *et al.*, 2009 ▸). Also, operation and background sound in a VR scene can enhance a user’s sense of presence and help them recognize operation sequences and risks (Lu & Davis, 2016 ▸). To achieve high fidelity of cryoEM operations, we first focused on the perception of visual and auditory fidelity. For auditory fidelity, we have embedded the recorded sound of physical instruments in *CryoVR* and play the recordings during the corresponding virtual operations. For visual fidelity, we should not only make the virtual devices and laboratory environment look real, but must also take special care of small objects that are often involved in cryoEM operations, for example holding a grid with tweezers. To achieve realism, we rendered high-level details for these objects in close-up views. However, when the user works on other tasks, these details of small objects will be unnecessary and could waste computing resources that may hinder fluency. We thus make *CryoVR* adaptively display the level of detail of the virtual objects to ensure both visual fidelity and high fluency.

## 6. Dissemination and usability test

To determine the usability and effectiveness of *CryoVR*, we conducted several rounds of usability testing. Usability testing has proved be one of the main methods to evaluate virtual reality applications (Fussell *et al.*, 2019 ▸). With the opinions from cryoEM device operators, we can measure the influence of *CryoVR* in cryoEM training procedures. We can also detect usability problems in *CryoVR* to propose improvements and solutions that optimize the user experience.

In March 2022, the National Center of CryoEM Access and Training (NCCAT) held a two-day workshop on cryoEM operation. 16 biological researchers participated in the workshop. We were invited to demonstrate *CryoVR* in the workshop. We invited these 16 participants to experience the four single-particle cryoEM VR training modules. We then interviewed and collected their opinions about *CryoVR*. These questions are from the IGROUP questionnaire template. Participants gave very positive feedback (Table 1 ▸). The results show the fact that users are actively engaged in operating cryoEM devices in the virtual environment (Q3, 4.5). They feel the devices are realistic (Q1, 3.89) and feel presence in a cryoEM laboratory (Q2, 3.56). The instructions shown in the VR scenes are clear and effective. They agree that *CryoVR* is an effective training tool (Q4, 4.03; Q5, 4.56), are willing to use *CryoVR* in their future training and will recommend *CryoVR* to other people (Q7, 4.06; Q8, 4.61).

Due to the limitations of the hardware (VR goggles and controllers), the appearance of cryoEM devices in VR still has room for improvement (Q1 and Q2).

*CryoVR* has been released to the community for anyone to freely download from https://www.purdue.edu/cryoVR. The *CryoVR* website also includes additional information such as VR hardware/software requirements, installation instruments, source-code availability on GitHub and a gallery of videos of *CryoVR* modules *etc*. Over the past few years of developing *CryoVR*, we have also had multiple chances to disseminate our work to the cryoEM community through training workshops at a variety of cryoEM events and conferences. We have received much constructive feedback from the users:

The VR training system was really well programmed and easy to navigate.

I feel like overall it is a pretty good way to explain a procedure that I had no idea what it was for. I think that the technology was consistent and easy to use, I had no troubles following the on screen directions and the indicator lights that showed where to place things. I imagine that it is also a lot more attention grabbing than a training video, simulates the ‘hands on experience’.

To assess the value to students with limited experience of cryo-EM, we disseminated our HTC Vive version of the *CryoVR* Training mode and Exam mode to students in the cryoEM class and collected their feedback. Except for minor bugs, their comments were generally positive and they agreed that it is a good preview of the procedures, especially for students without any hands-on cryo-EM experience. Here are some of the comments from them:

Overall, it is good and can [be] used for educational purpose especially for the new users like us.

This is a helpful preview for all the procedures before [we] practice it on the real instrument.

## 7. Conclusion

Our *CryoVR* training system provides a unique cryoEM training experience of learning by doing in a virtual environment to acquaint users with instrument interfaces, operation procedures and manipulation skills before starting hands-on training with physical cryoEM instruments. It enables multi-modal learning by integrating text, visual and audio cues and other training materials such as relevant video clips of instruments and SOPs to create a holistic training approach. It is a fun and engaging experience that incentivizes users to practice more. It is a self-paced training system in a VR environment that users can use to practice anywhere and anytime rather than having to be in the facility and relying on limited trainer and instrument availability. This capability is particularly useful in situations where in-person contact is severely limited, as in the current COVID-19 pandemic. Furthermore, it simulates hazard consequences of incorrect operations to allow trainees to virtually experience dangerous situations from a first-person perspective, and trains users to properly handle and recover from a hazardous situation.

## Figures and Tables

**Table 1:** Average and SD scores of the IGROUP questionnaire

Question	Average	SD
Q1. Realism for cryoEM devices	3.89	0.76
Q2. Sense of presence	3.56	0.92
Q3. Engagement	4.50	0.51
Q4. If VR experience can help hands-on training	4.03	0.85
Q5. The effectiveness of *CryoVR*	4.56	0.62
Q6. Clarity of instructions	4.33	1.03
Q7. Willingness to use *CryoVR* in future training	4.06	0.87
Q8. Willingness to recommend *CryoVR* to others	4.61	0.50
